# Chromosomal structural rearrangements implicate long non-coding RNAs in rare germline disorders

**DOI:** 10.1007/s00439-024-02693-y

**Published:** 2024-07-26

**Authors:** Rebecca E. Andersen, Ibrahim F. Alkuraya, Abna Ajeesh, Tyler Sakamoto, Elijah L. Mena, Sami S. Amr, Hila Romi, Margaret A. Kenna, Caroline D. Robson, Ellen S. Wilch, Katarena Nalbandian, Raul Piña-Aguilar, Christopher A. Walsh, Cynthia C. Morton

**Affiliations:** 1 Division of Genetics and Genomics and Manton Center for Orphan Diseases, Boston Children’s Hospital, Boston, MA USA; 2 Harvard Medical School, Boston, MA USA; 3 Broad Institute of MIT and Harvard, Cambridge, MA USA; 4 Department of Obstetrics and Gynecology, Brigham and Women’s Hospital, Boston, MA USA; 5 Harvard College, Cambridge, MA USA; 6 Division of Genetics, Department of Genetics, Brigham and Women’s Hospital, Boston, MA USA; 7 Department of Pathology, Brigham and Women’s Hospital, Boston, MA USA; 8 Department of Otolaryngology, Boston Children’s Hospital, Boston, MA USA; 9 Department of Radiology, Boston Children’s Hospital, Boston, MA USA; 10 Howard Hughes Medical Institute, Boston, MA USA; 11 University of Manchester, Manchester Center for Audiology and Deafness, Manchester, UK

## Abstract

In recent years, there has been increased focus on exploring the role the non-protein-coding genome plays in Mendelian disorders. One class of particular interest is long non-coding RNAs (lncRNAs), which has recently been implicated in the regulation of diverse molecular processes. However, because lncRNAs do not encode protein, there is uncertainty regarding what constitutes a pathogenic lncRNA variant, and thus annotating such elements is challenging. The Developmental Genome Anatomy Project (DGAP) and similar projects recruit individuals with apparently balanced chromosomal abnormalities (BCAs) that disrupt or dysregulate genes in order to annotate the human genome. We hypothesized that rearrangements disrupting lncRNAs could be the underlying genetic etiology for the phenotypes of a subset of these individuals. Thus, we assessed 279 cases with BCAs and selected 191 cases with simple BCAs (breakpoints at only two genomic locations) for further analysis of lncRNA disruptions. From these, we identified 66 cases in which the chromosomal rearrangements directly disrupt lncRNAs. In 30 cases, no genes of any other class aside from lncRNAs are directly disrupted, consistent with the hypothesis that lncRNA disruptions could underly the phenotypes of these individuals. Strikingly, the lncRNAs *MEF2C-AS1* and *ENSG00000257522* are each disrupted in two unrelated cases. Furthermore, we experimentally tested the lncRNAs *TBX2-AS1* and *MEF2C-AS1* and found that knockdown of these lncRNAs resulted in decreased expression of the neighboring transcription factors *TBX2* and *MEF2C*, respectively. To showcase the power of this genomic approach for annotating lncRNAs, here we focus on clinical reports and genetic analysis of seven individuals with likely developmental etiologies due to lncRNA disruptions.

**Supplementary Information:**

The online version contains supplementary material available at 10.1007/s00439-024-02693-y.

## Introduction

Only ~ 2% of the human genome is represented in protein-coding messenger RNAs (mRNAs) (Mattick and Amaral 2022). The non-protein-coding genome comprises a diverse array of elements including those that transcribe long non-coding RNAs (lncRNAs), transcripts of at least 200 nucleotides in length that are not translated into proteins (Kopp and Mendell 2018). Current transcriptome annotations (Frankish et al. 2021) suggest that there are nearly 20,000 human lncRNAs, rivaling the number of protein-coding genes, and the expression of many of these lncRNAs is highly regulated. However, the biological roles of most lncRNAs remain to be determined, which is made challenging by the fact that lncRNAs can carry out a wide variety of different functions. For instance, some lncRNAs can act in *trans* to affect diverse biological processes at distant sites, whereas many other lncRNAs have been shown to act in *cis* to regulate target genes in a manner that depends upon the location from which the lncRNA is transcribed (Gil and Ulitsky 2020). *Cis*-acting lncRNAs can exist in many different genomic configurations with respect to their target gene, such as overlapping in the sense or antisense direction, sharing a promoter region, or encompassing enhancer elements that regulate the target gene (Statello et al. 2021; Ferrer and Dimitrova 2024). In some cases, the lncRNA locus exists at a substantial distance from its target gene when considering the linear nucleotide position, however it can be brought into proximity with its target by the three-dimensional chromatin conformation. *Cis*-acting lncRNAs can also carry out their roles in diverse ways, including through RNA-dependent mechanisms as well as through functions that do not require the lncRNA transcript itself, such as the act of transcription of the lncRNA locus (Gil and Ulitsky 2020). By regulating the expression of neighboring genes, *cis*-acting lncRNAs can play critical roles in modulating key biological processes.

As a testament to the importance of lncRNAs in normal development, deletions of certain lncRNAs have led to lethal phenotypes in mice as well as abnormal development of the neocortex, lung, gastrointestinal tract, and heart (Sauvageau et al. 2013; Feyder and Goff 2016; Andersen et al. 2019; Mattick et al. 2023). In addition, lncRNAs have been implicated in cancer and shown to affect cell division, metabolism, and tumor-host interactions. Thus, lncRNAs are essential to maintaining proper cellular homeostasis (Statello et al. 2021). A prior observation of a variant in a lncRNA causing human disease in a Mendelian fashion is a 27–63 kb deletion of a locus that encompasses a lncRNA upstream of the engrailed-1 gene (*EN1*) (MIM: 131290), which resulted in congenital limb abnormalities even though *EN1* itself was not disrupted (Allou et al. 2021). Most recently, a pre-print manuscript (Ganesh et al. 2024) reported three individuals with deletions in *CHASERR*, a lncRNA proximal to *CHD2* (MIM: 602119), a protein-coding gene that causes developmental and epileptic encephalopathy. Intriguingly, disruption of *CHASERR* leads to increased expression of *CHD2* in *cis*, leading to a distinct clinical presentation compared to individuals with *CHD2* haploinsufficiency.

Given the importance of lncRNAs to gaining a better understanding of developmental biology and improving clinical diagnoses, it is important to develop a better functional assessment of lncRNAs in the human genome. However, the methods of annotating protein-coding genes are not typically applicable to non-coding genes due to their fundamental differences (Mattick et al. 2023). For instance, single nucleotide variants (SNVs) can result in nonsense mutations that prematurely terminate proteins, and indels can cause translational frame shifts that alter the entire downstream amino acid sequence of a protein. However, because lncRNAs do not encode proteins, it is unclear what affect if any such mutations may have on lncRNAs. The study of lncRNAs remains to be elucidated with regard to definitive consensus over what constitutes a pathogenic lncRNA variant.

A prior landmark development in expanding a focus on DNA beyond protein-coding genes to the 3D genome was the discovery of topologically associated domains (TADs). TADs are megabase-sized genomic segments partitioning the genome into large regulatory units with frequent intra-domain chromatin interactions but relatively rare inter-domain interactions (Lupiáñez et al. 2015). Conserved across different cell types and species, they are considered crucial for spatiotemporal gene expression patterns. Topological boundary regions (TBRs) block interactions between adjacent TADs, and TBR disruption by chromosomal structural rearrangements can result in rewiring of genomic regulators leading to abnormal clinical phenotypes. Rigorous interpretation of clinical phenotypes requires assessment of the boundaries of TADs following a chromosomal structural rearrangement because complex phenotypes may be dissected from rearrangements that reposition lncRNAs with respect to the relevant protein-coding region. Such rearrangements can also disrupt or reposition important non-coding regulatory elements such as enhancers, and thus careful consideration of these various possibilities is required (D’haene and Vergult 2021). Along with lncRNAs, chromatin conformation, enhancer-associated chromatin modifications (e.g., H3K4me1 and H3K27Ac), and experimentally validated enhancer elements should all be analyzed when interpreting non-coding structural variants.

The Developmental Genome Anatomy Project (DGAP) (Higgins et al. 2008) and similar projects have historically explored balanced chromosomal rearrangements to establish possible relationships between genotypes and phenotypes through identifying nucleotide-level breakpoints via Sanger sequencing. Individuals with such rearrangements represent natural gene disruptions and dysregulations, and their chromosomal rearrangements can serve as ideal signposts for annotating the human genome. Unlike for protein-coding genes, it is hard to predict the pathogenicity of lncRNA variants because they are not translated and consequently frameshift and nonsense mutations may not disrupt their function. Here, we employ a foundational approach in human genetics using chromosomal rearrangements to interrogate potential phenotypic impacts of disrupted lncRNAs and their genomic repositioning resulting in dysregulation. Both disruption and dysregulation of lncRNAs therefore may increase the diagnostic yield of developmental disorders. We venture to make a call out to cytogeneticists to employ further the power of chromosomal rearrangements in yet another opportunity to contribute to annotating the genome, recognizing that there are many patients and families who still await diagnoses.

## Methods

### Human subjects

Study ID numbers are a consecutive alphanumeric list that are not known outside of the research group. The Partners HealthCare System Internal Review Board (IRB) gave ethical approval for this work under protocol number 1999P003090.

### Breakpoint mapping

Genomic DNA from DGAP probands was sequenced to identify chromosomal breakpoints at nucleotide-level according to the previously published protocol (Talkowski et al. 2011; Hanscom and Talkowski 2014). Sanger sequencing results were aligned to the human genome using the UCSC Genome Browser BLAT tool (Kent 2002). Breakpoints were also compiled from previous publications (Talkowski et al. 2012b; Redin et al. 2017; Lowther et al. 2022). Breakpoint positions were converted from earlier genome builds to hg38 using the UCSC Genome Browser LiftOver tool (Kent et al. 2002).

### Additional genetic analyses

To ensure that the patient phenotypes could not be attributed to other variants aside from the chromosomal rearrangements, whole exome sequencing was performed for a subset of cases by the Genomics Platform at the Broad Institute of MIT and Harvard (Cambridge, MA). Sequencing libraries were prepared from sample DNA (250 ng input) using the Twist Bioscience exome (~ 35 Mb target) assay (San Francisco, CA), which were then sequenced (150 bp paired end) on the Illumina NovaSeq platform (Illumina, San Diego, CA) to generate a coverage of > 85% of the target region at 20X read depth or greater. Sequencing data for each of the samples was processed through an internal pipeline using the BWA aligner for mapping to the human genome (GRCh38/hg38) and variant calling was performed using the Genome Analysis Toolkit (GATK) HaplotypeCaller package. Variants were then annotated and assessed for pathogenicity using the Seqr software (Pais et al. 2022). Variants with > 1% MAF were filtered out and variants in genes with an association with disease were prioritized for analysis. No candidate variants associated with the phenotypes were found.

### TAD analysis and visualization of chromatin interactions

TAD boundary positions previously identified by Dixon and colleagues (Dixon et al. 2012) were converted from hg18 to hg38 using the UCSC Genome Browser LiftOver tool (Kent et al. 2002). BEDTools was used to identify TADs that included DGAP breakpoints (Quinlan and Hall 2010). The USCS Genome Browser (Kent et al. 2002) was used to display these TAD regions. Along with the genes within these regions, we also display chromatin interactions identified through micro-C studies from H1-hESCs (Krietenstein et al. 2020). Furthermore, we display layered tracks showing the enhancer-associated chromatin modifications H3K4me1 and H3K27Ac based on ChIP-seq analyses (ENCODE Project Consortium 2012). These ChIP-seq datasets were generated from seven human cell lines representing a variety of cell types (GM12878 lymphoblastoid cells, H1-hESCs, human skeletal muscle myoblasts, human umbilical vein endothelial cells, K562 chronic myelogenous leukemia cells, normal human epidermal keratinocytes, and normal human lung fibroblasts) by the Bernstein Lab at the Broad Institute. We also show VISTA-validated enhancer elements that demonstrated reproducible patterns of activity through in vivo transgenic mouse assays (Visel et al. 2007).

### Temporal bone computerized tomography (CT)

Axial temporal bone CT without contrast for Fig. S1A-B consisted of helical images with the following parameters: Discovery STE system (General Electric Healthcare, Waukesha, WI); 0.625 mm slice thickness, effective mAs of 17, mA of 158, rotation time of 2161 milliseconds, pitch of 0.5625 and kvp of 140. Images were viewed in a plane parallel to that of the horizontal semicircular canal. Coronal reformatted images were obtained in a plane perpendicular to the axial images at 0.74 mm thickness.

Axial temporal bone CT without contrast for Fig. S1C-D consisted of helical images with the following parameters: Discovery STE system (General Electric Healthcare, Waukesha, WI); 0.625 mm slice thickness, effective mAs of 27, mA of 246, rotation time of 2161 milliseconds, pitch of 0.5625 and kvp of 100. Images were viewed in a plane parallel to that of the horizontal semicircular canal. Coronal reformatted images were obtained in a plane perpendicular to the axial images at 0.70 mm thickness.

### Knockdown experiments

Target lncRNAs were knocked down in the human Lenti-X™ 293T Cell Line (Takara # 632180) using Silencer^®^ Select siRNAs from ThermoFisher Scientific (see Table S4 for siRNA details). When available, siRNA sequences from previous publications were used (Zhang et al. 2016; Modi et al. 2023). For each sample, 12.5pmol of siRNA was reverse-transfected into 200,000 cells using 3uL of Lipofectamine™ RNAiMAX Transfection Reagent (ThermoFisher Scientific #13778150) in a final culture volume of 250uL. After 24 h, RNA was collected using TRI Reagent and stored at -80 °C. RNA was later purified using the Direct-zol RNA Microprep Kit (Zymo Research # R2061). For gene expression analysis, cDNA synthesis was performed using SuperScript™ IV VILO™ Master Mix (ThermoFisher Scientific #11756050), and digital droplet PCR (ddPCR) was performed using QX200™ ddPCR™ EvaGreen Supermix (Bio-Rad #1864033), the Automated Droplet Generator (Bio-Rad #1864101), and the QX200 Droplet Reader (Bio-Rad #1864003). For each sample, expression was normalized using the internal control gene *HPRT1* (Anderson et al. 2015). See Table S4 for ddPCR primer details.

## Results

### Identification of human subjects with disrupted lncRNAs

We evaluated 279 cases of balanced chromosomal abnormalities and selected 191 cases with resolved breakpoints indicating a “simple” rearrangement (i.e., breakpoints at only two genomic locations and no significant genomic imbalance) for further analysis (Table S1). Using the most recent Human Gencode Reference, Release 45, GRCh38.p14 (Frankish et al. 2021), we then identified 66 cases in which at least one breakpoint overlapped a lncRNA (Table S2 and Table S3). Overall, 79 unique lncRNAs were directly disrupted in these cases, and four lncRNAs including *MEF2C-AS1* and *ENSG00000257522* were each disrupted in two unrelated individuals. In 30 of the cases, no genes of any other class aside from lncRNAs were directly disrupted by the breakpoints. In this report, we present seven cases disrupting five different lncRNAs as examples of the potential value of assessing lncRNAs as diagnostic etiologies (Table 1).

**Table 1:** Details regarding the breakpoints and the disrupted genes for the cases highlighted in this manuscript. Genomic coordinates refer to GRCh38/hg38

Subject_ID	break	chr	break_start	break_end	gene_start	gene_end	strand	gene_name	gene_type
DGAP148	1	chrX	39882591	39882592	.	.	.	.	.
DGAP148	2	chr11	127040509	127040509	127021736	127172363	+	ENSG00000255087	lncRNA
DGAP191	1	chr5	89411065	89411070	88883328	89466398	+	MEF2C-AS1	lncRNA
DGAP191	2	chr7	94378248	94378255	94278680	94395608	-	ENSG00000285090	lncRNA
DGAP218	1	chr5	24272189	24272193	.	.	.	.	.
DGAP218	2	chr5	89105026	89105031	88883328	89466398	+	MEF2C-AS1	lncRNA
DGAP245	1	chr3	36927650	36927959	36826819	36945057	-	TRANK1	protein_coding
DGAP245	2	chr14	29276109	29276117	28936889	29372406	+	ENSG00000258028	lncRNA
DGAP245	2	chr14	29276109	29276117	29210986	29392048	-	ENSG00000257522	lncRNA
DGAP353	1	chr14	65855359	65855360	.	.	.	.	.
DGAP353	2	chr17	61393812	61393841	61361668	61400243	-	ENSG00000267131	lncRNA
DGAP353	2	chr17	61393812	61393841	61393455	61411555	-	TBX2-AS1	lncRNA
DGAP355	1	chr3	181488756	181489591	180989762	181836880	+	SOX2-OT	lncRNA
DGAP355	2	chr9	74712100	74713321	.	.	.	.	.
NIJ1	1	chr8	78898169	78898172	78805293	78956082	+	MITA1	lncRNA
NIJ1	2	chr14	29296328	29296331	28936889	29372406	+	ENSG00000258028	lncRNA
NIJ1	2	chr14	29296328	29296331	29210986	29392048	-	ENSG00000257522	lncRNA

### *TBX2-AS1* is a candidate lncRNA for an association with hearing loss

The proband DGAP353 was diagnosed during gestation when her 24-year-old healthy mother underwent amniocentesis following an abnormal maternal serum screen for an elevated risk for trisomy 21. An apparently balanced translocation was detected in the female fetus between the long arms of chromosomes 14 and 17. Parental chromosome analyses revealed maternal inheritance and apparent structural identity to the maternal t(14;17) rearrangement. The daughter’s G-banded karyotype is described as 46,XX,t(14;17)(q24.3;q23)mat and the mother’s karyotype is 46,XX,t(14;17)(q24.3;q23). No clinical abnormalities were observed in the fetus and the pregnancy was continued. The daughter began developing signs of hearing loss at around the age of 10 years, and her hearing loss was found to be primarily sensorineural with a conductive element. By the age of 12 years, surgery was performed to rectify the conductive abnormalities, but the sensorineural hearing loss remained. The mother began wearing hearing aids at around the age of 40 years after a gradual decline in hearing for an unspecified time period. Both the daughter and her mother were otherwise healthy, typical of nonsyndromic deafness of unknown genetic etiology. Computerized tomography (CT) imaging of the temporal bones of the mother and daughter revealed abnormalities such as unusually small sinus tympani and narrowing of the round and oval windows (Fig. S1).

**Fig. 1:**
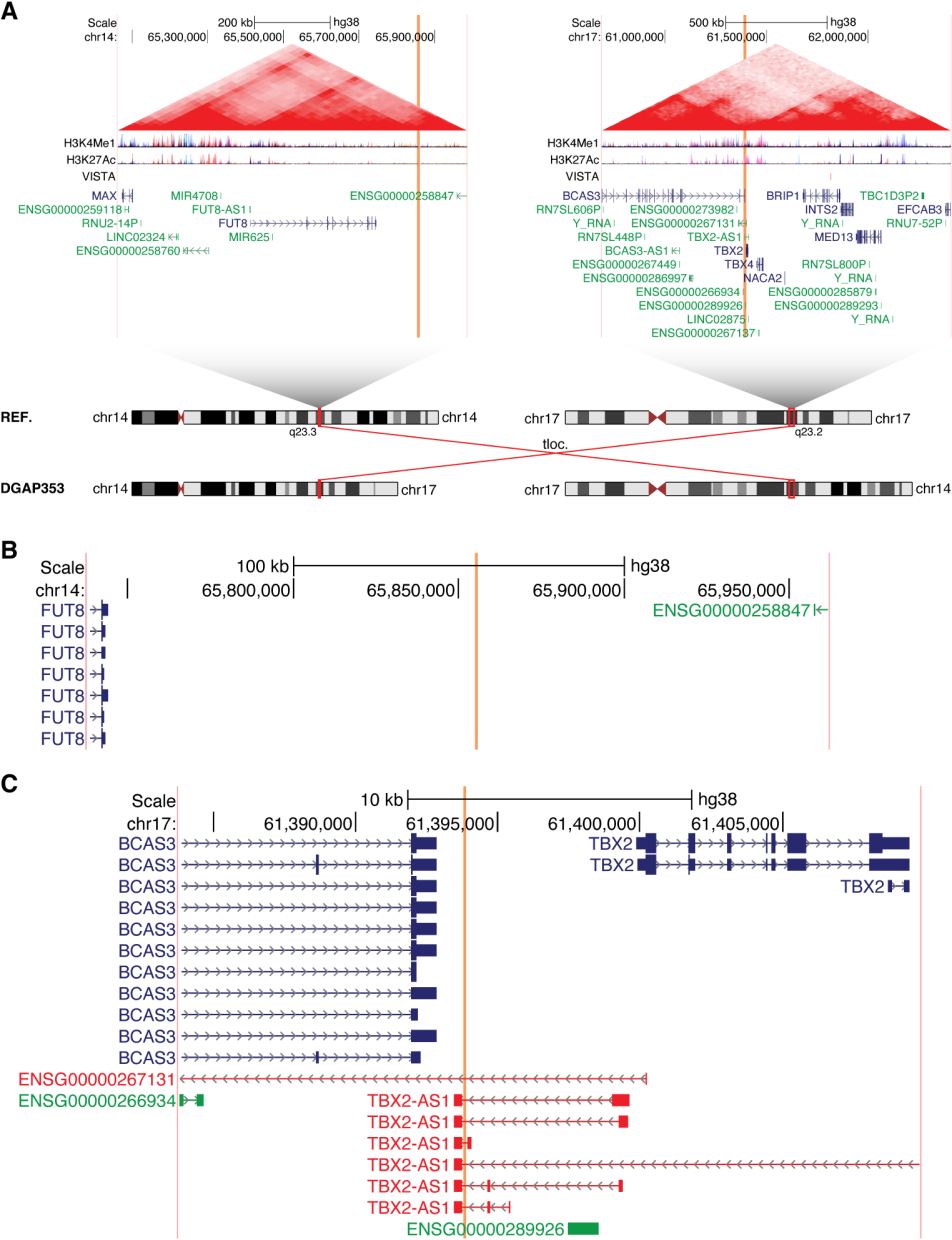
The lncRNA *TBX2-AS1* is a candidate for an association with hearing loss. (**A**) Chromosome diagrams depict the translocation between 14q23.3 and 17q23.2 in DGAP353. Above, TADs containing the breakpoints are shown, with the breakpoint positions indicated by vertical orange bars and the edges of the region shown in vertical pink bars. TAD borders were defined in (Dixon et al. 2012). Triangular contact maps display micro-C data indicating chromatin conformation (Krietenstein et al. 2020). H3K4Me1 and H3K27Ac tracks depict enhancer-associated chromatin modifications (ENCODE Project Consortium 2012). The VISTA track shows experimentally validated enhancer elements (Visel et al. 2007). Protein-coding genes are shown in blue and non-coding genes in green, with a single isoform depicted per gene. (**B**) Expanded view of the genomic region surrounding the 14q23.3 breakpoints in DGAP353. (**C**) Expanded view of the genomic region surrounding the 17q23.2 breakpoints in DGAP353. The directly disrupted lncRNA *TBX2-AS1* is highlighted in red. *ENSG00000267131* has been identified as an isoform of *TBX2-AS1* by LNCipedia (Volders et al. 2019)

Both mother and daughter (DGAP353) harbor a translocation between chromosomes 14 and 17 with a 7 base-pair (bp) insertion of DNA of non-templated origin at the breakpoint in the der(17) chromosome (Fig. 1A). Following revision of suggested nomenclature (Ordulu et al. 2014), the next-generation cytogenetic nucleotide level research rearrangement is described below in a single string.

46,XX,t(14;17)(q24.3;q23)mat.seq[GRCh38] t(14;17)(14pter→14q23.3(+)(65,855,3{58–60})::17q23.2(+)(61,393,84{1–3})→17qter;17pter→17q23.2(+)(61,393,812)::TATATAC::14q23.3(+)(65,855,359)→14qter)mat

The DGAP353 breakpoints do not overlap any genes on chromosome 14 (Fig. 1B); however, this translocation results in the direct disruption of the lncRNA *TBX2-AS1* from chromosome 17 (Fig. 1C). The Gencode annotation also lists the lncRNA *ENSG00000267131* as a separate gene that is disrupted by these breakpoints, however this has been identified as an isoform of *TBX2-AS1* (called *TBX2-AS1:3*) on the basis of shared exonic sequences by LNCipedia (Volders et al. 2019) (Fig. S2). While little is known regarding the biological role of *TBX2-AS1*, particularly in the context of hearing, the orthologous mouse lncRNA (*2610027K06Rik*) has been detected in the cochlear and vestibular sensory epithelium of embryonic and postnatal mice (identified as “XLOC_007930”) (Ushakov et al. 2017). Using the Gene Expression Analysis Resource (gEAR) portal (Orvis et al. 2021), we further found that while *Tbx2-as1* is detected in supporting cell types (pillar and Deiters cells), it is predominantly expressed by sensory inner hair cells, as determined through cell-type-specific RNA-seq (Liu et al. 2018). Thus, the expression pattern of *Tbx2-as1* is consistent with the finding that the hearing loss demonstrated by DGAP353 and her mother is primarily sensorineural.

The lncRNA gene *TBX2-AS1* exists in a divergent configuration with the transcription factor *TBX2* (MIM: 600747). Divergent lncRNAs have transcriptional start sites (TSSs) within 5 kb of another gene and are transcribed in the opposite direction in a “head-to-head” configuration. Importantly, the DGAP353 translocation does not disrupt the shared *TBX2/TBX2-AS1* promoter region, but rather removes the 3’ end of *TBX2-AS1*, suggesting a potential lncRNA-dependent mechanism. Divergent lncRNAs have generally been associated with positive regulation of their neighboring gene, particularly when the neighbor is a transcription factor (Luo et al. 2016; Wang et al. 2020). Some divergent lncRNAs have also been found to modulate the downstream functions of the protein generated by the neighboring gene. Thus, knowledge regarding the function of the protein-coding member of a divergent pair can provide insights into the potential biological role of the lncRNA partner.

Intriguingly, *TBX2* has previously been linked with hearing and inner ear development. In mice, *Tbx2* has been associated with otocyst patterning in inner ear morphogenesis, as mouse models in which *Tbx2* was conditionally knocked out exhibit cochlear hypoplasia (Kaiser et al. 2021). Previous studies have also shown that deletions encompassing *TBX2* and *TBX2-AS1* are found in individuals with hearing loss, albeit in conjunction with other deleted genes (Ballif et al. 2010; Nimmakayalu et al. 2011; Schönewolf-Greulich et al. 2011). In addition, a recent study has shown that *Tbx2* is required for inner hair cell and outer hair cell differentiation, demonstrating that it is a master regulator of hair cell fate (García-Añoveros et al. 2022). Therefore, we suggest that the translocation disrupting *TBX2-AS1* in DGAP353 and her mother may lead to altered expression or function of *TBX2*, ultimately resulting in the phenotype of hearing loss.

### Recurrent disruptions of the lncRNA *MEF2C-AS1* in individuals with neurological phenotypes

We additionally identified two cases, DGAP191 and DGAP218, with chromosomal rearrangements that disrupt the lncRNA *MEF2C-AS1*. The next-generation cytogenetic nucleotide level research rearrangements are described below in single strings.

*DGAP191*: 46,XY,t(5;7)(q14.3;q21.3)dn.seq[GRCh38] t(5;7)(5pter→5q14.3(+)(89,411,06{3–5})::7q21.3(+)(94,378,2{48–50})→7qter;7pter→7q21.3(+)(94,378,25{3–5})::5q14.3(+)(89,411,07{0–2})→5qter)dn

*DGAP218*: 46,XX,inv(5)(p12q13.1)dn.seq[GRCh38] inv(5)(pter→p14.2(+)(24,272,19{3})::q14.3(-)(89,105,02{6})→p14.2(-)(24,272,189)::TATTTATATGACAAG::q14.3(+)(89,105,031)→qter)dn

**Fig. 2:**
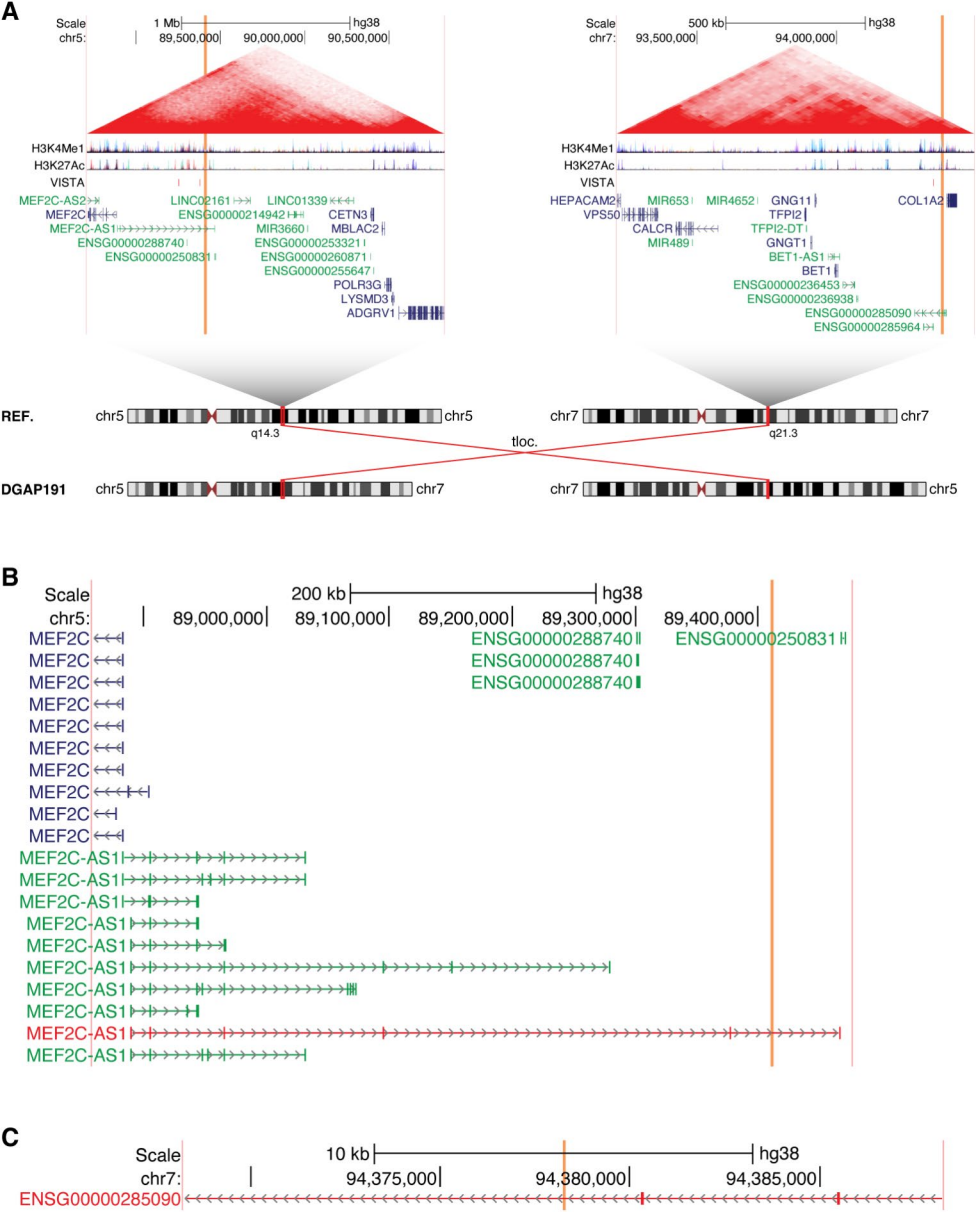
The lncRNA *MEF2C-AS1* is disrupted in multiple individuals with neurological phenotypes, as shown here for DGAP191. (**A**) Chromosome diagrams depict the translocation between 5q14.3 and 7q21.3 in DGAP191. Above, TADs containing the breakpoints are shown, with the breakpoint positions indicated by vertical orange bars and the edges of the region shown in vertical pink bars. TAD borders were defined in (Dixon et al. 2012). Triangular contact maps display micro-C data indicating chromatin conformation (Krietenstein et al. 2020). H3K4Me1 and H3K27Ac tracks depict enhancer-associated chromatin modifications (ENCODE Project Consortium 2012). The VISTA track shows experimentally validated enhancer elements (Visel et al. 2007). Protein-coding genes are shown in blue and non-coding genes in green, with a single isoform depicted per gene. (**B**) Expanded view of the genomic region surrounding the 5q14.3 breakpoints in DGAP191. The directly disrupted lncRNA *MEF2C-AS1* is highlighted in red. (**C**) Expanded view of the genomic region surrounding the 7q21.3 breakpoints in DGAP191. The directly disrupted lncRNA *ENSG00000285090* is highlighted in red

**Fig. 3:**
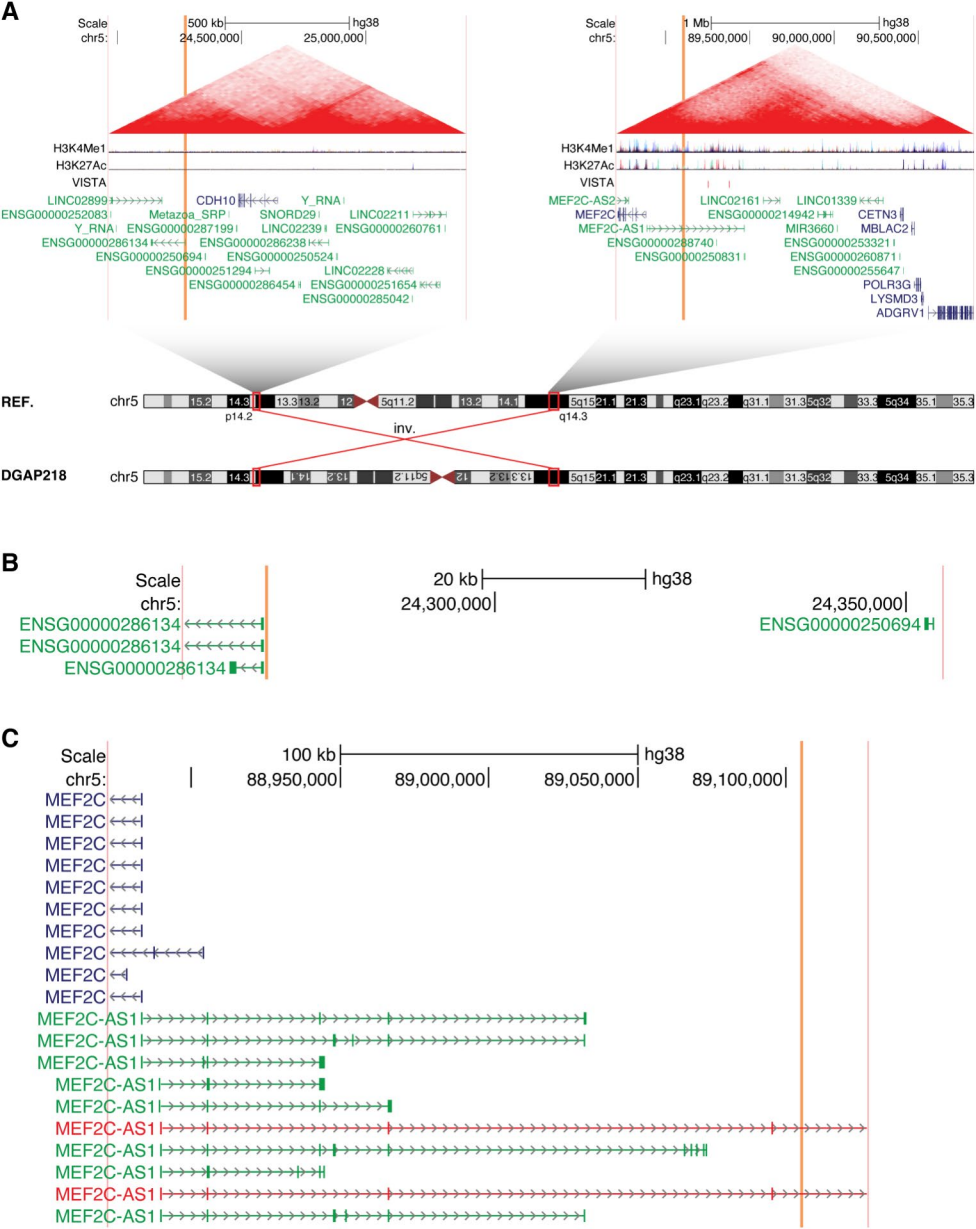
The lncRNA *MEF2C-AS1* is disrupted in multiple individuals with neurological phenotypes, as shown here for DGAP218. (**A**) Chromosome diagrams depict the inversion between 5p14.2 and 5q14.3 in DGAP218. Above, TADs containing the breakpoints are shown, with the breakpoint positions indicated by vertical orange bars and the edges of the region shown in vertical pink bars. TAD borders were defined in (Dixon et al. 2012). Triangular contact maps display micro-C data indicating chromatin conformation (Krietenstein et al. 2020). H3K4Me1 and H3K27Ac tracks depict enhancer-associated chromatin modifications (ENCODE Project Consortium 2012). The VISTA track shows experimentally validated enhancer elements (Visel et al. 2007). Protein-coding genes are shown in blue and non-coding genes in green, with a single isoform depicted per gene. (**B**) Expanded view of the genomic region surrounding the 5p14.2 breakpoints in DGAP218. (**C**) Expanded view of the genomic region surrounding the 5q14.3 breakpoints in DGAP218. The directly disrupted lncRNA *MEF2C-AS1* is highlighted in red

In both cases, the 5q14.3 breakpoints directly disrupt the lncRNA *MEF2C-AS1*. In DGAP191, the 7q21.3 breakpoints additionally overlap the lncRNA *ENSG00000285090*, but no protein-coding genes are directly disrupted (Fig. 2). In DGAP218, *MEF2C-AS1* is the only gene of any class that is directly disrupted (Fig. 3).

We previously reported both of these individuals as part of a larger set of cases with breakpoints in 5q14.3 (Redin et al. 2017). This region is of particular interest due to 5q14.3 microdeletion syndrome, which is characterized by neurological phenotypes including intellectual disability and epilepsy (Zweier and Rauch 2012). This syndrome is now recognized to be driven by decreased expression of *MEF2C* (MIM: 600662), either through direct disruption of *MEF2C* or due to distal mutations (Zweier and Rauch 2012). Indeed, when we previously described DGAP191 and DGAP218 (Redin et al. 2017), we noted that their phenotypes were similar to individuals with direct *MEF2C* disruptions. Furthermore, we determined that levels of *MEF2C* expression were reduced by ~ 30% in lymphoblastoid cell lines from both DGAP191 and DGAP218 (Redin et al. 2017); however, no mention was made of *MEF2C-AS1*. Recent studies have further elucidated the functional effects of altering *MEF2C* or its topological organization (Mohajeri et al. 2022), but the potential role of *MEF2C-AS1* remains unclear.

While there is still little known regarding the function of *MEF2C-AS1*, it has recently been found that overexpression of *MEF2C-AS1* can increase the levels of *MEF2C* in human cervical cancer cell lines by serving as a microRNA sponge (Guo et al. 2022). Interestingly, *MEF2C-AS1* is transcribed through multiple putative enhancers of *MEF2C* (D’haene et al. 2019), providing another potential mechanism for this lncRNA to regulate expression of its neighboring gene, as has previously been described for lncRNAs such as *Bendr* (Engreitz et al. 2016) and *Uph* (Anderson et al. 2016). Thus, for DGAP191 and DGAP218 we now propose that the disruption of *MEF2C-AS1* leads to decreased expression of *MEF2C*, resulting in neurological phenotypes.

### The lncRNA *ENSG00000257522* is recurrently disrupted in individuals with microcephaly

**Fig. 4:**
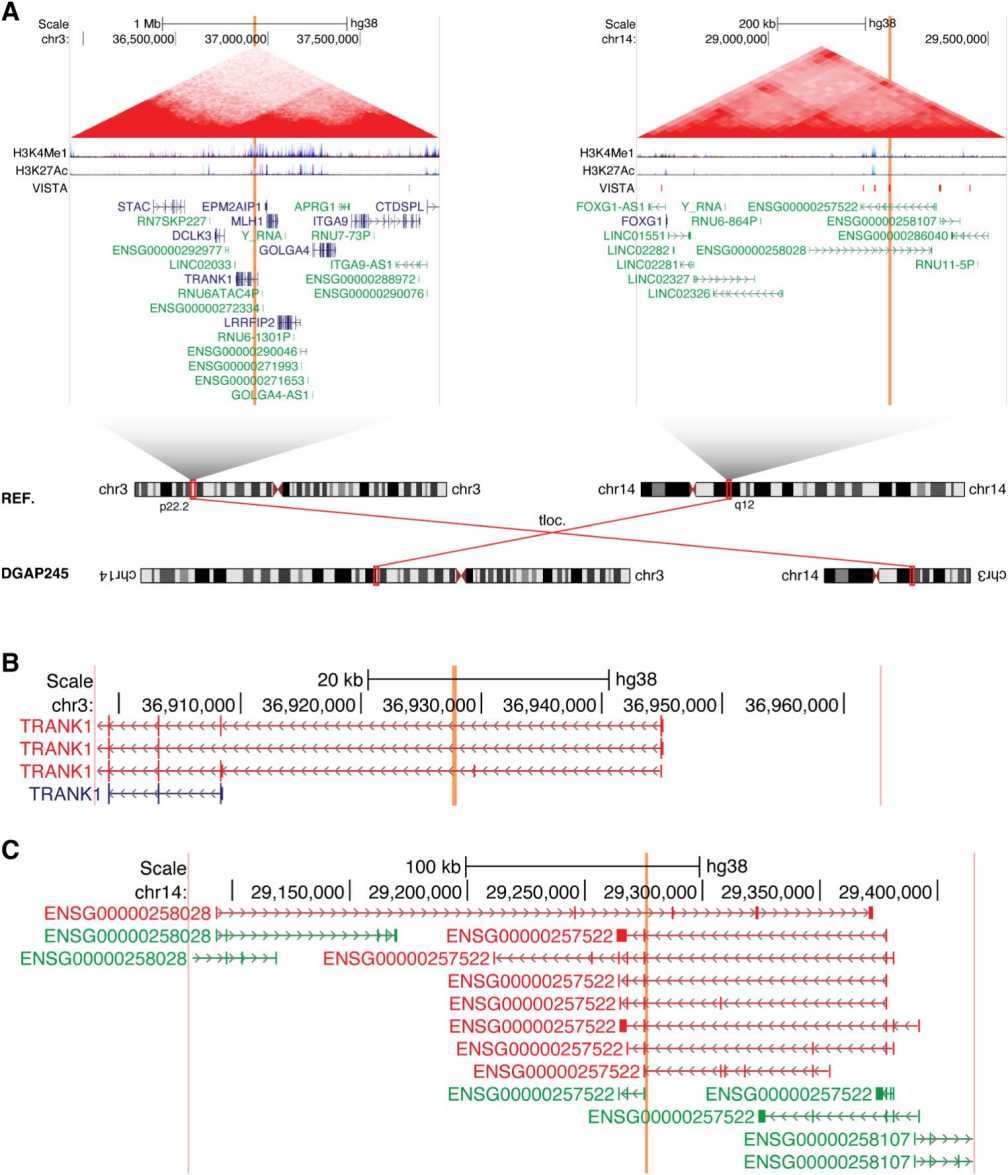
The lncRNA *ENSG00000257522* is disrupted in multiple individuals with microcephaly, as shown here for DGAP245. (**A**) Chromosome diagrams depict the translocation between 3p22.2 and 14q12 in DGAP245. Above, large regions containing the breakpoints are shown, with the breakpoint positions indicated by vertical orange bars and the edges of the region shown in vertical pink bars. The region shown surrounding 14q12 is a TAD, with its borders previously defined in (Dixon et al. 2012). No TAD was defined surrounding 3p22.2, so instead the region including 1 Mb on either side of the breakpoints is displayed. Triangular contact maps display micro-C data indicating chromatin conformation (Krietenstein et al. 2020). H3K4Me1 and H3K27Ac tracks depict enhancer-associated chromatin modifications (ENCODE Project Consortium 2012). The VISTA track shows experimentally validated enhancer elements (Visel et al. 2007). Protein-coding genes are shown in blue and non-coding genes in green, with a single isoform depicted per gene (**B**) Expanded view of the genomic region surrounding the 3p22.2 breakpoints in DGAP245. (**C**) Expanded view of the genomic region surrounding the 14q12 breakpoints in DGAP245. The directly disrupted lncRNAs *ENSG00000258028* and *ENSG00000257522* are highlighted in red

**Fig. 5:**
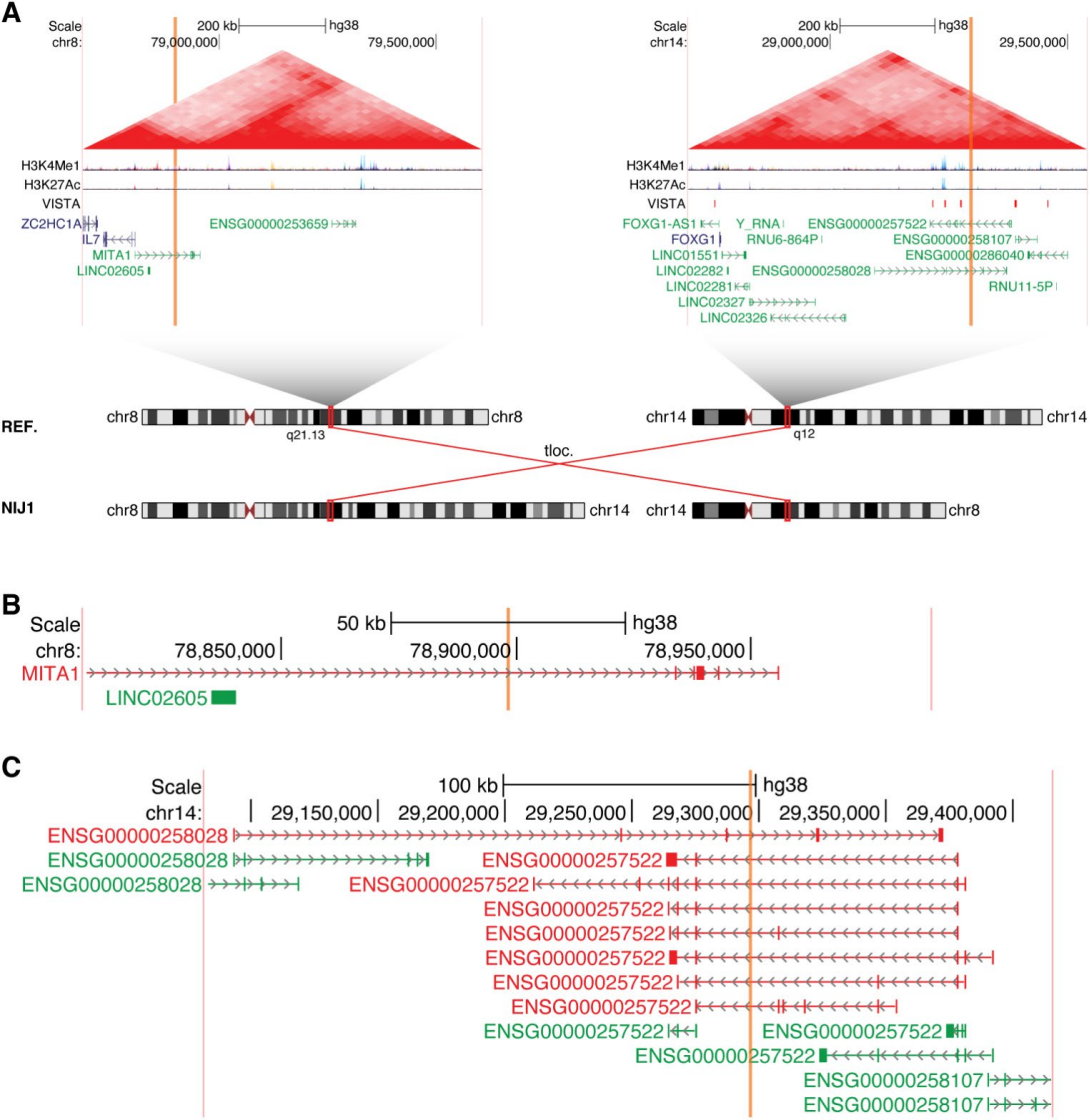
The lncRNA *ENSG00000257522* is disrupted in multiple individuals with microcephaly, as shown here for NIJ1. (**A**) Chromosome diagrams depict the translocation between 8q21.13 and 14q12 in NIJ1. Above, TADs containing the breakpoints are shown, with the breakpoint positions indicated by vertical orange bars and the edges of the region shown in vertical pink bars. TAD borders were defined in (Dixon et al. 2012). Triangular contact maps display micro-C data indicating chromatin conformation (Krietenstein et al. 2020). H3K4Me1 and H3K27Ac tracks depict enhancer-associated chromatin modifications (ENCODE Project Consortium 2012). The VISTA track shows experimentally validated enhancer elements (Visel et al. 2007). Protein-coding genes are shown in blue and non-coding genes in green, with a single isoform depicted per gene. (**B**) Expanded view of the genomic region surrounding the 8q21.13 breakpoints in NIJ1. (**C**) Expanded view of the genomic region surrounding the 14q12 breakpoints in NIJ1. The directly disrupted lncRNAs *ENSG00000258028* and *ENSG00000257522* are highlighted in red

Our analysis further identified two cases, DGAP245 and NIJ1, with chromosomal rearrangements that disrupt the lncRNA *ENSG00000257522* (Figs. 4 and 5). These individuals exhibit shared phenotypes including microcephaly and defects of the corpus callosum (Table S1). The next-generation cytogenetic nucleotide level research rearrangements are described below in single strings.

*DGAP245*: 46,XY,t(3;14)(p23;q13)dn.seq[GRCh38] t(3:14)(3qter→3p22.2(-)(36,927,959)::CATTTGTTCAAATTTAGTTCAAATGA::14q12(+)(29,276,117)→14qter;14pter→14q12(+)(29,276,10{8–9})::3p22.2(-)(36,927,6{49–50})→3pter)dn

*NIJ1*: 46,XX,t(8;14)(q21.2;q12)dn.seq[GRCh38] t(8;14)(8pter→8q21.12(+)(78,898,16{9})::14q12(+)(29,296,33{1})→14qter;14pter→14q12(+)(29,296,328)::AAAT::8q21.12(+)(78,898,172)→8qter)dn

In both cases, the 14q12 breakpoints directly disrupt the lncRNA *ENSG00000257522* as well as the overlapping antisense lncRNA *ENSG00000258028*. In DGAP245, the 3p22.2 breakpoints additionally disrupt the protein-coding gene *TRANK1* (MIM: 619316) (Fig. 4B), however this gene is not predicted to be haploinsufficient (pHaplo = 0.29) (Collins et al. 2022) and it has not been implicated in any human phenotypes by OMIM. In NIJ1, the 8q21.12 breakpoints disrupt the lncRNA *MITA1* (Fig. 5B). Given that the only shared disruptions between these cases are to the lncRNAs *ENSG00000257522* and *ENSG00000258028*, we focused on these for further analysis.

Using the GTEx database (Lonsdale et al. 2013), we found that *ENSG00000258028* is not readily detected in neural tissue, and thus it is unlikely to cause the patient phenotypes. In contrast, *ENSG00000257522* is primarily expressed in neural tissue (Fig. S3A), suggesting that it could play an important neurological role. Moreover, *ENSG00000257522* exists within the same TAD as the protein-coding gene *FOXG1* (MIM: 164874), which similarly exhibits a predominantly neural expression pattern (Fig. S3B). Disruptions in *FOXG1* have been associated with a variant of Rett syndrome (MIM: 613454) (Ariani et al. 2008) as well as *FOXG1* syndrome (Kortüm et al. 2011). Core phenotypes of these syndromes include microcephaly and corpus callosum defects, implicating *FOXG1* dysregulation as the underlying genetic etiology in DGAP245 and NIJ1. Thus, we sought to identify potential regulatory elements that could be disrupted by the chromosomal rearrangements in these cases, and found three regions with prominent H3K4me1 chromatin modification (Fig.S3C), which is associated with enhancer activity (ENCODE Project Consortium 2012). Notably, one of these regions also exhibited H3K27Ac modification, which is also associated with enhancer activity (ENCODE Project Consortium 2012). Furthermore, these three regions each include VISTA enhancers that have been demonstrated to drive reporter expression in neural tissue in vivo in transgenic mice (hs566, hs1539, and hs1168) (Visel et al. 2007), and thus these regions exert experimentally validated enhancer activity.

Strikingly, all three of these enhancers exist within the lncRNA *ENSG00000257522*. While the most distal enhancer is partially disrupted by the breakpoints in DGAP245, the other two enhancers remain in the appropriate position relative to *FOXG1*. In NIJ1, all three of the enhancers are proximal to the breakpoints and are not separated from *FOXG1*. Thus, these enhancers are not directly disrupted by the chromosomal rearrangements, and instead their activity could be impaired due to the disruption of the lncRNA in which they are embedded. Indeed, transcription of lncRNAs through enhancers is a well-documented mechanism through which lncRNAs can regulate gene expression (Statello et al. 2021). Thus, we propose that the lncRNA *ENSG00000257522* regulates the expression of *FOXG1* through its effects on the embedded enhancers. This is consistent with previous findings that several lncRNAs function to modulate the expression of transcription factors and that this tight regulation is essential for maintaining proper functions of the transcription factors, particularly for pioneer factors such as *FOXG1* (Ferrer and Dimitrova 2024).

Further supporting this, we also identified an individual with a complex *de novo* rearrangement that similarly disrupts *ENSG00000257522*. This individual, DGAP246, exhibits consistent phenotypes including microcephaly (Redin et al. 2017). The complex rearrangement in DGAP246 consists of 14 pairs of breakpoints, including eight breakpoints in 14q12. Overall, this results in the direct disruption of the lncRNA *ENSG00000257522* while leaving the two most proximal enhancer elements in their correct position relative to *FOXG1* (Fig. S3C). Taken together, these three cases implicate the lncRNA *ENSG00000257522* in the regulation of *FOXG1*. Additionally, previous studies have reported several individuals with *FOXG1* syndrome that harbor disruptions in this region, including a translocation in “Patient 1” that directly disrupts *ENSG00000257522* (Mehrjouy et al. 2018). Similarly, a recent case report described another individual with *FOXG1* syndrome whose balanced translocation had breakpoints mapping within the *ENSG00000257522* lncRNA (Craig et al. 2020). Thus, we propose that disruptions of this lncRNA can cause phenotypes including microcephaly and defects of the corpus callosum, consistent with *FOXG1* syndrome.

### Potential regulation of *KIRREL3* by its neighboring lncRNA *ENSG00000255087*

We previously described DGAP148 as an individual with a neurodevelopmental disorder including attention deficits and difficulty with spatial coordination (Talkowski et al. 2012b). We have recently received updated information from the referring clinical geneticist indicating that this individual is in overall good health but continues to receive medication for attention-deficit/hyperactivity disorder (ADHD). She was not able to complete regular high school, however she works as a helper in a veterinary clinic. While she lives with her father, she is autonomous for tasks of everyday living, including meals, laundry, exercise, and driving. She is also described as very sociable.

**Fig. 6:**
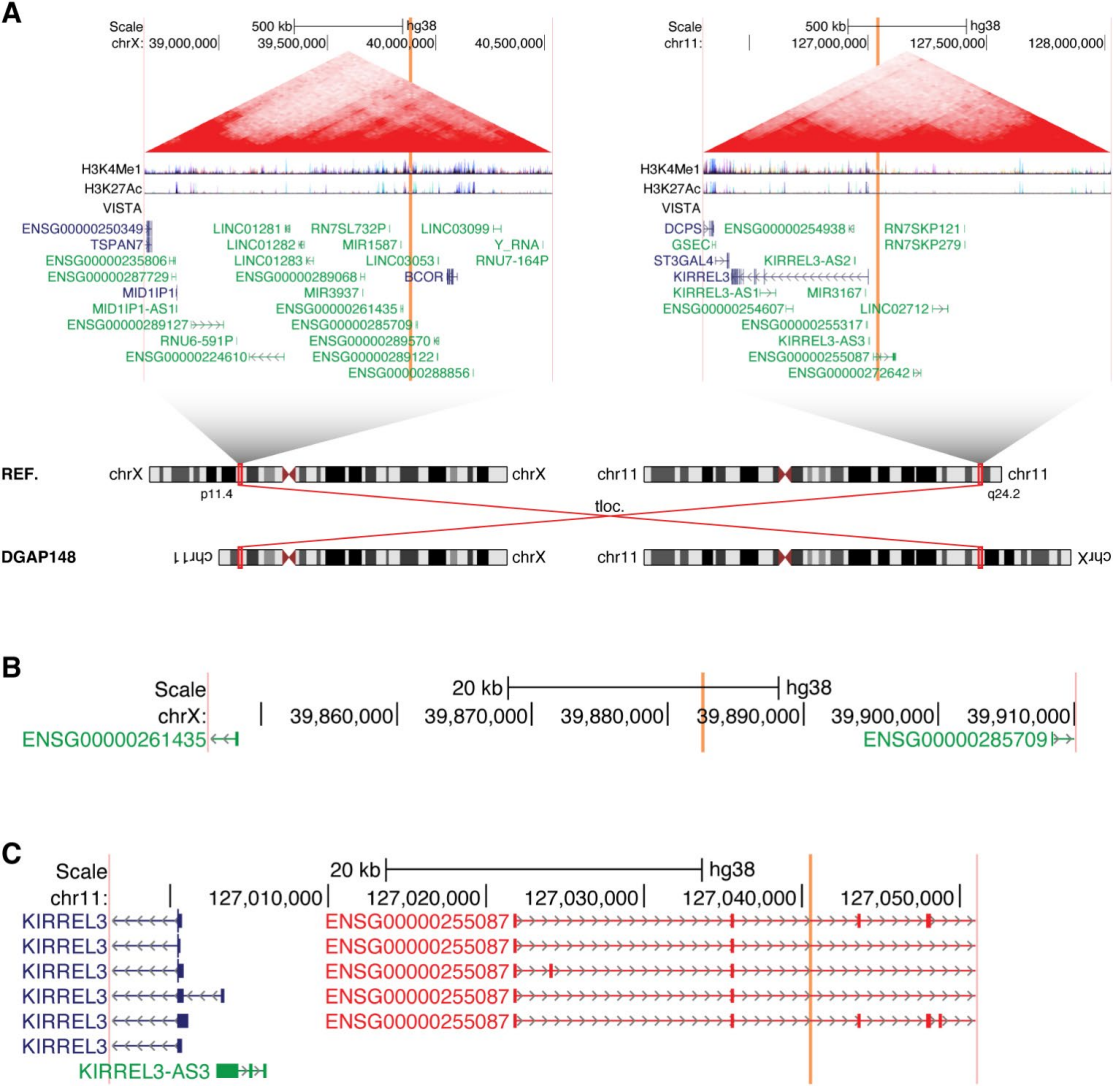
Disruption of the lncRNA *ENSG00000255087* was identified in an individual with a neurodevelopmental disorder. (**A**) Chromosome diagrams depict the translocation between Xp11.4 and 11q24.2 in DGAP148. Above, TADs containing the breakpoints are shown, with the breakpoint positions indicated by vertical orange bars and the edges of the region shown in vertical pink bars. TAD borders were defined in (Dixon et al. 2012). Triangular contact maps display micro-C data indicating chromatin conformation (Krietenstein et al. 2020). H3K4Me1 and H3K27Ac tracks depict enhancer-associated chromatin modifications (ENCODE Project Consortium 2012). The VISTA track shows experimentally validated enhancer elements (Visel et al. 2007). Protein-coding genes are shown in blue and non-coding genes in green, with a single isoform depicted per gene. (**B**) Expanded view of the genomic region surrounding the Xp11.4 breakpoints in DGAP148. (**C**) Expanded view of the genomic region surrounding the 11q24.2 breakpoints in DGAP148. The directly disrupted lncRNA *ENSG00000255087* is highlighted in red

DGAP148 has a *de novo* translocation (Fig. 6A), and the next-generation cytogenetic nucleotide level research rearrangement is described below in a single string.

46,X,t(X;11)(p11.2;q23.3)dn.seq[GRCh38] t(X;11)(Xqter→Xp11.4(-)(39,882,592)::TCACTGTACAG::11q24.2(+)(127,040,509)→11qter;11pter→11q24.2(+)(127,040,509)::CTC::Xp11.4(-)(39,882,591)→Xpter)dn

No genes are directly disrupted by the Xp11.4 breakpoints (Fig. 6B), whereas the 11q24.2 breakpoints disrupt the lncRNA *ENSG00000255087* (Fig. 6C).

At the time of our initial report of DGAP148 (Talkowski et al. 2012b), we were unaware of the lncRNA *ENSG00000255087*, which still lacks any PubMed publications. However, *ENSG00000255087* is approximately 20 kb upstream of the protein-coding gene *KIRREL3* (MIM: 607761), which has been associated with neurodevelopmental phenotypes including attention deficits (Ciaccio et al. 2021; Querzani et al. 2023). We previously found that expression of *KIRREL3* was reduced by ~ 30% in DGAP148 (Talkowski et al. 2012b), but the potential mechanism underlying this was unclear. Upon reanalyzing this case and determining that the lncRNA *ENSG00000255087* is directly disrupted, we used the GTEx database (Lonsdale et al. 2013) to assess the expression of *ENSG00000255087*. We find that *ENSG00000255087* is predominantly expressed in neural tissue (Fig. S4A), similar to the *KIRREL3* expression pattern (Fig. S4B) and consistent with a potential neurodevelopmental role. Considering that the translocation in DGAP148 directly disrupts *ENSG00000255087* and that DGAP148 exhibits decreased *KIRREL3* levels, we suggest that the lncRNA *ENSG00000255087* is a candidate for regulating expression of its neighboring gene *KIRREL3*. Furthermore, we propose that disruption of *ENSG00000255087* can thus lead to the neurodevelopmental phenotypes described for DGAP148.

### The lncRNA *SOX2-OT* is implicated in an individual with epilepsy and autism spectrum disorder

**Fig. 7:**
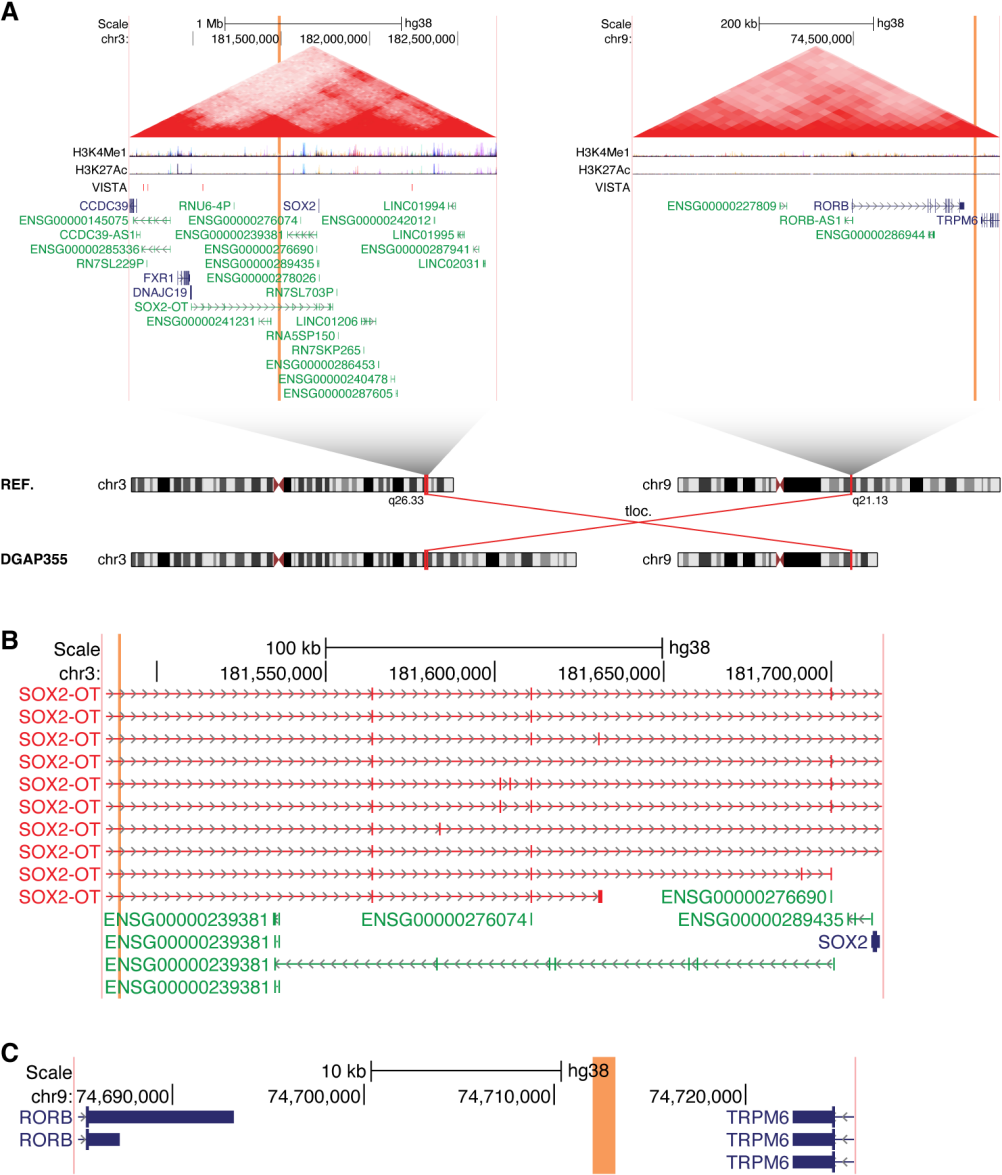
The lncRNA *SOX2-OT* is implicated in an individual with epilepsy and autism spectrum disorder. (**A**) Chromosome diagrams depict the translocation between 3q26.33 and 9q21.13 in DGAP355. Above, TADs containing the breakpoints are shown, with the breakpoint positions indicated by vertical orange bars and the edges of the region shown in vertical pink bars. TAD borders were defined in (Dixon et al. 2012). Triangular contact maps display micro-C data indicating chromatin conformation (Krietenstein et al. 2020). H3K4Me1 and H3K27Ac tracks depict enhancer-associated chromatin modifications (ENCODE Project Consortium 2012). The VISTA track shows experimentally validated enhancer elements (Visel et al. 2007). Protein-coding genes are shown in blue and non-coding genes in green, with a single isoform depicted per gene. (**B**) Expanded view of the genomic region surrounding the 3q26.33 breakpoints in DGAP355. The directly disrupted lncRNA *SOX2-OT* is highlighted in red. (**C**) Expanded view of the genomic region surrounding the 9q21.13 breakpoints in DGAP355

DGAP355 is a nonverbal individual with global developmental delay, autism spectrum disorder (ASD), seizures, and epilepsy, whose mother has a history of three miscarriages. This individual has a *de novo* translocation between chromosomes 3 and 9 (Fig. 7A), and the next-generation cytogenetic nucleotide level research rearrangement, as solved by liWGS, is described below in a single string.

46,XX,t(3;9)(q26.3;q21.1)dn.seq[GRCh38] t(3;9)(3pter→3q26.33(+)(181,488,756)::9q21.13(+)(74,713,321)→9qter;9pter→9q21.13(+)(74,712,100)::3q26.33(+)(181,489,591)→3qter)dn

The 3q26.33 breakpoints occur within the lncRNA *SOX2-OT* (MIM: 616338) (Fig. 7B), which is the only gene directly disrupted by this translocation (Fig. 7C). *SOX2-OT* consists of dozens of isoforms that together span a nearly 850 kb genomic region. The protein-coding gene *SOX2* (MIM: 184429) exists entirely within an intron of *SOX2-OT* and is transcribed in the same direction. *SOX2* is a transcription factor that serves as a crucial regulator of the potency and self-renewal capacity of several progenitor cell types (Arnold et al. 2011), and in particular *SOX2* is known to play important roles in neural progenitor cells (Graham et al. 2003). *SOX2-OT* exhibits a similar expression pattern to *SOX2*, with both genes primarily expressed in neural tissue (Fig. S5). Recently, *SOX2-OT* has been found to affect *SOX2* expression in varying ways in different contexts (Shahryari et al. 2015; Knauss et al. 2018; Li et al. 2020; Yin et al. 2020). Thus, we propose that the translocation in DGAP355 that disrupts the lncRNA *SOX2-OT* may lead to dysregulated expression of *SOX2*, resulting in neurodevelopmental phenotypes including ASD and epilepsy.

### Knockdown of the lncRNAs *TBX2-AS1* and *MEF2C-AS1* results in decreased expression of their neighboring transcription factors

We sought to directly test the roles of implicated lncRNAs using the experimentally tractable human 293 cell line. We found that the lncRNAs *TBX2-AS1* and *MEF2C-AS1* were both expressed in this cell line, so we selected these candidates for further studies. Based on the genetic analyses described above, we proposed that these lncRNAs may regulate the expression of their neighboring transcription factors (*TBX2* and *MEF2C*, respectively). Thus, we performed siRNA-mediated knockdown experiments to determine whether depleting these lncRNA transcripts leads to altered expression levels of their neighbors.

**Fig. 8:**
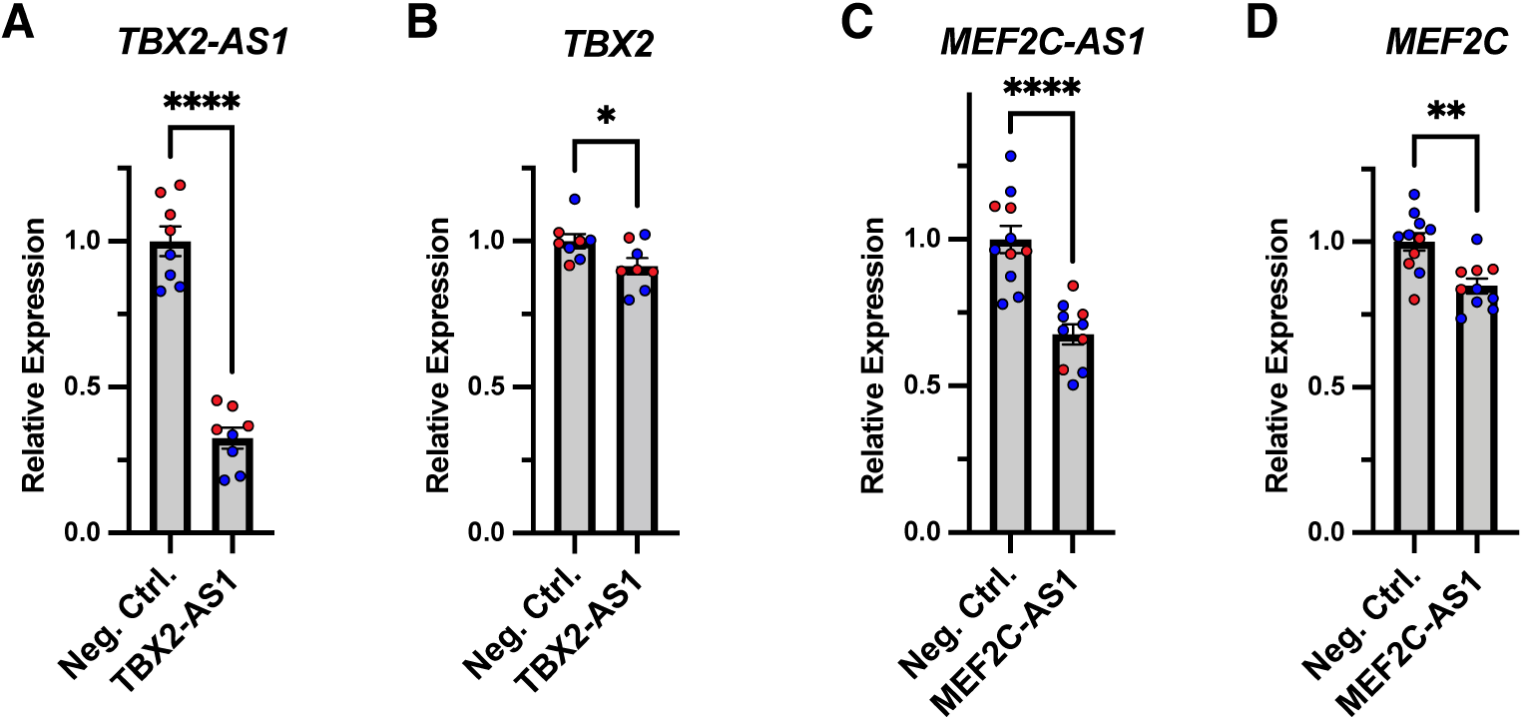
Knockdown of the lncRNAs *TBX2-AS1* and *MEF2C-AS1* results in decreased expression of their neighboring genes. (**A**) Relative expression of *TBX2-AS1* upon transfection with siRNAs targeting *TBX2-AS1*, as compared to negative control (Neg. Ctrl.) siRNAs. **** *p* < 0.0001. (**B**) Relative expression of *TBX2* upon transfection with siRNAs targeting *TBX2-AS1*. * *p* = 0.0367. (**C**) Relative expression of *MEF2C-AS1* upon transfection with siRNAs targeting *MEF2C-AS1*. **** *p* < 0.0001. (**D**) Relative expression of *MEF2C* upon transfection with siRNAs targeting *MEF2C-AS1*. ** *p* = 0.0013. All experiments were performed in human Lenti-X™ 293T cultures with two separate negative control and targeting siRNAs, with the results from siRNA #1 shown in blue and from siRNA #2 in red. Statistical analyses were performed using unpaired t tests

To knock down *TBX2-AS1*, we transfected 293 cultures with two separate siRNAs that had demonstrated strong efficiency in a previous study (Modi et al. 2023). After 24 h, we collected RNA from these samples and found that the siRNAs led to a 67.5% decrease in *TBX2-AS1* expression (*p* < 0.0001) (Fig. 8A). Furthermore, this resulted in a small (8.6%) but statistically significant (*p* = 0.0367) decrease in the expression of *TBX2* (Fig. 8B). Given the variety of *TBX2-AS1* transcript isoforms (Fig. S2), it was not possible to target all of these isoforms with the siRNAs, which could contribute to the relatively small change in *TBX2*. We similarly used two custom siRNAs to knock down *MEF2C-AS1* in 293s, which resulted in a 32.4% decrease in *MEF2C-AS1* levels (*p* < 0.0001) (Fig. 8C). Moreover, this led to a 15.1% decrease in *MEF2C* (*p* = 0.0013) (Fig. 8D). These results demonstrate that depleting the transcripts of these lncRNAs is alone sufficient to decrease the expression of their neighboring transcription factors. Many lncRNAs have been found to “fine-tune” the expression of transcription factors, and even small changes in transcription factor levels can lead to substantial phenotypic consequences (Ferrer and Dimitrova 2024); thus, these experiments further implicate the lncRNAs *TBX2-AS1* and *MEF2C-AS1* in rare germline disorders.

### Several lncRNAs are directly disrupted in DGAP cases

Additional DGAP cases in which lncRNAs are directly disrupted are listed in Table S2. These lncRNAs warrant further consideration, particularly for cases in which no other genes are directly disrupted. Given the abundance of lncRNAs throughout the human genome, it is not rare for chromosomal rearrangements to disrupt lncRNAs, and yet this class of gene has remained largely overlooked. As updated human genome annotations continue to include new lncRNAs, it is increasingly likely to identify lncRNAs disrupted by chromosomal rearrangements. The cases described here emphasize the importance of carefully considering such disrupted lncRNAs when evaluating potential genetic etiologies underlying patient conditions.

## Discussion

By virtue of their noncoding nature, it is difficult to assess the pathogenicity of lncRNA variants based on standards for protein-coding genes. As such, we propose a novel framework to implicate lncRNAs based on chromosomal rearrangements that disrupt the lncRNA. Importantly, chromosomal rearrangements can also separate non-coding regulatory elements from the genes they modulate, complicating the analysis of non-coding structural variants (D’haene and Vergult 2021). While all of the cases we discuss involve BCAs that directly disrupt lncRNAs, it remains possible that other non-coding elements such as enhancers may play a role in these cases as well. Thus, for all cases we have included data depicting chromatin conformation (Krietenstein et al. 2020), the enhancer-associated chromatin modifications H3K4me1 and H3K27Ac (ENCODE Project Consortium 2012), and VISTA-validated enhancer elements (Visel et al. 2007). We also draw particular attention to these features when they are especially relevant to certain cases. Overall, however, we selected these cases because the BCAs have breakpoints that occur within lncRNAs. Taken together, the cases presented here strongly suggest that the disruption of lncRNAs can result in rare germline disorders.

We first propose that disruption of the lncRNA *TBX2-AS1* causes human disease in a Mendelian fashion in a familial case of hearing loss. This lncRNA is disrupted by a balanced chromosomal rearrangement that segregated with deafness/hard-of-hearing (DHH) from mother to daughter. Little is currently known about human *TBX2-AS1* other than that it is divergent to *TBX2*. It is not listed currently in OMIM, and available databases including gnomAD (Karczewski et al. 2020) and DECIPHER (Firth et al. 2009) cannot be used to determine the level of constraint in the human genome pool or the tolerance to haploinsufficiency for lncRNAs because these metrics are defined specifically for protein-coding genes. *TBX2*, however, has been linked to hearing and inner ear development, including through the identification of deletions encompassing *TBX2* and *TBX2-AS1* (among other genes) that were found in individuals with hearing loss (Ballif et al. 2010; Nimmakayalu et al. 2011; Schönewolf-Greulich et al. 2011).

In the DGAP353 proband presented herein, the breakpoint did not affect *TBX2* itself but interrupted *TBX2-AS1*. It has been shown that *TBX2* maps to the edge of a TAD and is linked to *TBX2-AS1* as a bi-directionally transcribed topological anchor point (tap)RNA (Amaral et al. 2018; Decaesteker et al. 2018). Expression levels of such lncRNAs have been found to be highly correlated with those of their nearest protein-coding genes, and this has also been observed between *TBX2* and *TBX2-AS1*, suggesting that *TBX2-AS1* and *TBX2* may be connected on a regulatory level (Wansleben et al. 2014; Decaesteker et al. 2018). Moreover, we found that siRNA-mediated knockdown of *TBX2-AS1* resulted in decreased expression of *TBX2* (Fig. 8B). It is also possible that *TBX2-AS1* could affect the function of the TBX2 protein, as has been demonstrated for other divergent lncRNAs (Rapicavoli et al. 2011; Vance et al. 2014; Pavlaki et al. 2018) and has recently been suggested for *TBX2-AS1* (Modi et al. 2023). Thus, we propose the lncRNA *TBX2-AS1* as a candidate for an association with hearing loss. To assess fully whether *TBX2-AS1* disruption is the causal agent for their hearing loss, a mouse model in which *Tbx2-as1* is knocked down while *TBX2* remains intact is under development. Additional cases of hearing loss with deleterious *TBX2-AS1* variants will be valuable to confirm the proposed association.

Furthermore, we describe recurrent disruptions of the lncRNAs *MEF2C-AS1* and *ENSG00000257522*, and we propose that these disruptions lead to altered expression of the neighboring transcription factors *MEF2C* and *FOXG1*, respectively. Both of these lncRNAs are transcribed through VISTA-validated enhancer elements (Visel et al. 2007), suggesting a potential mechanism through which these lncRNAs could affect the regulation of their neighboring transcription factors. Regarding *MEF2C*, we previously reported multiple cases with BCAs that separated *MEF2C* from putative enhancer elements (Redin et al. 2017). This set of cases included the two cases we focus on in this manuscript, DGAP191 and DGAP218. Thus, the separation of these distal enhancers from *MEF2C* could certainly contribute to the phenotypes of these individuals. However, we also demonstrated that knockdown of the lncRNA *MEF2C-AS1* is alone sufficient to cause decreased expression of *MEF2C* (Fig. 8D), suggesting that the direct disruption of this lncRNA also plays a role in the decreased expression of *MEF2C* that we previously reported in these individuals (Redin et al. 2017). With respect to *FOXG1*, we identified three VISTA-validated enhancer elements within the recurrently disrupted lncRNA *ENSG00000257522*. In one of the individuals (NIJ1), the rearrangement occurred distal to all three of these enhancers, leaving their positions unchanged relative to *FOXG1* (Fig.S3C). While even farther distal enhancers could also play a role, the recurrent disruptions of the lncRNA *ENSG00000257522* support its proposed involvement in the regulation of *FOXG1*.

Additionally, we suggest that the lncRNAs *ENSG00000255087* and *SOX2-OT* may regulate their neighboring genes *KIRREL3* and *SOX2*, respectively. These lncRNAs both exhibit expression patterns that are similar to their neighbors, with expression predominantly detected in neural tissue (Fig.S4-S5). While we previously reported decreased expression of *KIRREL3* upon disruption of *ENSG00000255087* in DGAP148 (Talkowski et al. 2012b), the function of this lncRNA has yet to be experimentally validated. Meanwhile, *SOX2-OT* has been shown to affect the expression of *SOX2* in different ways depending on the context (Shahryari et al. 2015; Knauss et al. 2018; Li et al. 2020; Yin et al. 2020), which would benefit from further characterization. Thus, future experiments in human neural models including monolayer cultures and three-dimensional organoids could be highly valuable for elucidating the biological roles of these lncRNAs.

Taken together, the seven cases we describe here implicate lncRNAs in several phenotypes. We have also identified many additional disrupted lncRNAs that warrant further investigation (TableS2). All of these lncRNAs were identified due to BCAs that would disrupt the lncRNA transcripts, so it is possible that these lncRNAs function through RNA-dependent mechanisms. Some of these lncRNAs are also transcribed through enhancer elements (e.g., *MEF2C-AS1* and *ENSG00000257522*) or other genes (e.g., *TBX2-AS1* and *SOX2-OT*), and the act of transcription itself may be an important part of how these lncRNAs could regulate neighboring gene expression, either in tandem with or independently from RNA-based functions (Andergassen and Rinn 2022). Thus, complete characterization of lncRNA mechanisms generally requires the combination of multiple approaches (Kopp and Mendell 2018), including RNA-targeting methods such as siRNAs or CRISPR-Cas13 (Konermann et al. 2018) as well as strategies to disrupt transcription or to replace the lncRNA transcript with a reporter (Andergassen and Rinn 2022).

Here, we used siRNAs to knock down two lncRNAs, which resulted in decreased expression of the neighboring transcription factors (Fig. 8). Many transcription factors are highly sensitive to small changes in expression, and minor disruptions can lead to meaningful phenotypic consequences (Ferrer and Dimitrova 2024). Thus, these experiments suggest that the RNA transcripts of these lncRNAs can play important biological roles; however, this does not preclude the possibility that they also function through RNA-independent mechanisms. We therefore propose that all of the lncRNAs discussed here warrant further experimental dissection in future studies. Human cell cultures, particularly from cell types that are relevant to the patient phenotypes, can provide a tractable model system. For lncRNAs that have mouse homologs such as *TBX2-AS1*, genetic mouse models can provide valuable insights into the roles of lncRNAs throughout development. Combining multiple model systems and experimental approaches will be especially powerful for characterizing intricate lncRNA functions.

The potential connections between the lncRNAs highlighted here and patient phenotypes were uncovered due to balanced chromosomal rearrangements in these loci; as such, we suggest that such rearrangements are an untapped resource to functionally annotate lncRNAs. We propose that geneticists pay special attention to potential dysregulation of lncRNAs in patients where balanced chromosomal rearrangements do not disrupt protein-coding genes in a manner consistent with the observed phenotypes. With an increasing number of chromosomal rearrangements mapped due to inexpensive whole genome sequencing and optical genome mapping, additional lncRNAs that underly developmental diseases await characterization.

### Electronic supplementary material

Below is the link to the electronic supplementary material.

Supplementary Material 1

Supplementary Material 2

Supplementary Material 3

Supplementary Material 4

Supplementary Material 5

Supplementary Material 6

Supplementary Material 7

Supplementary Material 8

Supplementary Material 9

Supplementary Material 10

## Data Availability

No datasets were generated or analysed during the current study.
