# Supplementary material for: Chromosomal structural rearrangements implicate long non-coding RNAs in rare germline disorders
Source: Hum Genet. 2024 Jul 26;143(7):921–38. doi: 10.1007/s00439-024-02693-y (PMC11294402; doi:10.1007/s00439-024-02693-y)
Supplement: Supplementary file 10 — Supplementary Material 10 [file 439_2024_2693_MOESM10_ESM.docx]

**Figure S1.** Computerized tomography (CT) images of the temporal bones reveal abnormalities in two individuals with hearing loss. A) Axial CT images of the right and left temporal bones of a 41-year-old female, the mother of DGAP353. While the inferior basal turns appeared normal (not shown), the upper basal and middle turns of each cochlea appear flattened (wide arrow in image 1). The round windows appear mildly narrow. The right cochlear aperture (short line in image 3) measured 1.5 mm transverse (TR) and the left measured 1.3 mm TR. There is variant anatomy of the internal auditory canals which appear mildly flared on axial images at the level of the porus acusticus, however normal in the coronal plane. The sinus tympani (posteromedial recess of the tympanic cavity) is unusually small bilaterally (short arrow in image 3). B) Reformatted coronal CT images of the right and left temporal bones of a 41-year-old female, the mother of DGAP353. The right oval window is mildly narrow in height (long arrow in image 1). The round window is also narrowed (short arrow in image 2). Note the mildly small upper cochlear turns (arrowhead in image 3). The left oval window is also mildly narrowed and opacified. The inferior osseous margin of the tympanic segment of the facial nerve canal is not clearly seen, raising concern for dehiscence at the level of the stenotic oval window (arrow in image 4). The subjacent round window appears normal in the coronal plane. C) Axial CT images of the temporal bones of a 12.5-year-old female, DGAP353. Imaging reveals normal upper cochlear turns. The cochlear aperture measures 1.6 mm on each side. The sinus tympani are unusually small bilaterally (short arrows). Note the normal right stapedial crura (long arrow in image 1). The left stapedial crura are closely approximated and indistinct (long arrow in image 2). D) Reformatted coronal images of the temporal bones of a 12.5-year-old female, DGAP353. These images reveal that the left oval window (arrow in image 1) and round window (arrow in image 2) are stenotic. The right sided oval and round windows were also slightly narrow (not shown).

**Figure S2.** Several different isoforms comprise the lncRNA *TBX2-AS1*. A) The DGAP353 breakpoint position is indicated by vertical orange bars and the edges of the region are shown in vertical pink bars. All directly disrupted transcripts in this region are shown in red, as annotated by the Human Gencode Reference, Release 45, GRCh38.p14 (Frankish et al. 2021). The neighboring gene *TBX2* is also shown for reference. For clarity, no other genes are shown. B) The same genomic region from panel A is shown with all *TBX2-AS1* isoforms annotated by LNCipedia (Volders et al. 2019). Directly disrupted transcripts are shown in red. *TBX2-AS1:3* corresponds to the transcript annotated as ENSG00000267131/ENST00000585765.1 by Gencode in panel A.

**Figure S3.** The lncRNA *ENSG00000257522* is transcribed through multiple enhancers that may regulate *FOXG1*. A) Expression of the lncRNA *ENSG00000257522* from the GTEx database (Lonsdale et al. 2013). B) Expression of the protein-coding gene *FOXG1* from the GTEx database (Lonsdale et al. 2013). C) Expanded view of the genomic region surrounding the 14q12 breakpoints in DGAP246, DGAP245, and NIJ1. Breakpoint positions are indicated by vertical orange bars, and the edges of the region are shown in vertical pink bars. H3K4Me1 and H3K27Ac tracks depict enhancer-associated chromatin modifications (ENCODE Project Consortium 2012). The VISTA track shows experimentally validated enhancer elements (Visel et al. 2007).

**Figure S4.** The lncRNA *ENSG00000255087* exhibits a similar pattern of expression to its neighboring gene *KIRREL3*. A) Expression of the lncRNA *ENSG00000255087* from the GTEx database (Lonsdale et al. 2013). B) Expression of the protein-coding gene *KIRREL3* from the GTEx database (Lonsdale et al. 2013).

**Figure S5.** The lncRNA *SOX2-OT* exhibits a similar pattern of expression to its overlapping gene *SOX2*. A) Expression of the lncRNA *SOX2-OT* from the GTEx database (Lonsdale et al. 2013). B) Expression of the protein-coding gene *SOX2* from the GTEx database (Lonsdale et al. 2013).

**Table S1.** Genetic and phenotypic details for all cases analyzed as part of this study. Genomic coordinates refer to GRCh38/hg38. Derivative A and Derivative B represent the chromosomal breakpoints listed in the order recommended by Ordulu et al. 2014.

**Table S2.** Details regarding the breakpoints and the directly disrupted genes for all 66 cases in which we identified a disrupted lncRNA. The first tab lists cases in which only lncRNAs are directly disrupted. The second tab lists cases in which lncRNAs are directly disrupted along with other genes. Genomic coordinates refer to GRCh38/hg38. The disruption of the lncRNA *RMST* in DGAP032 was previously reported in (Stamou et al. 2020). The disruption of the lncRNA *LINC00299* in DGAP162 was previously reported in (Talkowski et al. 2012a).

**Table S3.** Additional details for the cases in which only lncRNAs were directly disrupted. The first tab lists the nearest protein-coding gene to each disrupted lncRNA. The second tab lists all genes of any class within 100kb of the breakpoints, excluding the lncRNAs that are directly disrupted (see Table S2 for directly disrupted lncRNAs). Genomic coordinates refer to GRCh38/hg38.

**Table S4.** Details regarding the siRNAs (first tab) and the ddPCR primers (second tab) used for the knockdown experiments shown in Fig. 8.
